# Hybridized Phosphate with Ultrathin Nanoslices and Single Crystal Microplatelets for High Performance Supercapacitors

**DOI:** 10.1038/srep17613

**Published:** 2016-02-01

**Authors:** Yufeng Zhao, Zhaoyang Chen, Ding-Bang Xiong, Yuqing Qiao, Yongfu Tang, Faming Gao

**Affiliations:** 1 Key Laboratory of Applied Chemistry, Yanshan University, Qinhuangdao 066004, China; 2 State Key Laboratory of Metal Matrix Composites, Shanghai Jiao Tong University, Shanghai 200240, China

## Abstract

A novel hybridized phosphate is developed through a mild hydrothermal method to construct high performance asymmetric supercapacitor. Single layered (Ni,Co)_3_(PO_4_)_2_·8H_2_O nanoslices (∼1 nm) and single crystal (NH_4_)(Ni,Co)PO_4_·0.67H_2_O microplatelets are obtained through a template sacrificial method and dissolution recrystallization approach respectively in one step. This unique hybridized structure delivers a maximum specific capacitance of 1128 F g^−1^ at current density of 0.5 A g^−1^. The asymmetric supercapacitor (ASC) based on the hybrid exhibits a high energy density of 35.3 Wh kg^−1^ at low power density, and still holds 30.9 Wh kg^−1^ at 4400 W kg^−1^. Significantly, the ASC manifests very high cycling stability with 95.6% capacitance retention after 5000 cycles. Such excellent electrochemical performance could be attributed to the synergistic effect of the surface redox reaction from the ultrathin nanoslices and ion intercalation from the single crystal bulk structure. This material represents a novel kind of electrode material for the potential application in supercapacitors.

Supercapacitors also called electrochemical capacitors (ECs), have been of great interest, due to their high power density, rapid charge/discharge processes and long-term cycling stability^1^. However, the low energy density significantly hinders their practical application. Building an asymmetric supercapacitor is considered one of the most effective approaches to solve this problem. These asymmetric supercapacitors usually combine a battery-like pseudocapacitive electrode (as energy source) and a capacitive electrode (as power source) in one system, which can make full use of the different operation voltages of the electrode materials, and then increase the energy density and power density for the cell system^2^,^3^,^4^,^5^,^6^. In recent years, various battery-like materials with high pseudocapacitance have been investigated, among which transition metal compounds, including their oxides, hydroxides, and sulfides, have attracted significant attention^7^,^8^,^9^,^10^,^11^,^12^,^13^,^14^,^15^,^16^. Transition metal phosphates including ammonium transition metal phosphates, have been studied for decades and find broad applications in many industry fields^17^,^18^. Despite this, their application in the energy storage field has been less concerned until most recently^19^,^20^,^21^,^22^,^23^,^24^,^25^. Zhang *et al*.^25^ described a microwave-assisted one-pot oil-in-water emulsion method to synthesize a series of mesoporous Ni_x_Co_3-x_(PO_4_)_2_ hollow shell, which delivers a specific capacitance of 940 F g^−1^, indicating the potential application of nickel-cobalt phosphates in supercapacitors.

Generally, the pseudocapacitance of the battery type material is resulted from surface redox reaction, which depends heavily on the electrochemical active surface area of electrode materials^26^. Therefore, ultrathin layered electrode materials with most functional atoms exposed to the electrolyte can create sufficient electroactive sites, which unfortunately suffer from poor crystallinity with sacrificed electric conductivity and chemical stability. Bulk layered materials with fine crystallized structure, store energy through ion intercalation between atomic layers, on the other hand usually exhibit high rate performance and good stability^27^. Nevertheless the specific capacitance of the existing intercalation-type material is much lower than that from surface redox reactions. For instance, Pang *et al*.^24^ reported a well-crystalline NH_4_CoPO_4_·H_2_O with layered structure, which facilitates the redox reaction by the intercalation/deinterlation of hydroxyl ions. However its maximum specific capacitance only reached 369.4 F g^−1^. Therefore, it would be of great interest to construct a hybrid of ultrathin structure and bulk layered structure, which can combine the surface redox reaction and ion intercalation in one single system for the optimal capacitance behavior.

Herein we report the construction of a novel hybridized phosphate combining surface redox reaction and ion intercalation in one system for the first time. NH_4_-Co-Ni phosphates composed of (Ni,Co)_3_(PO_4_)_2_·8H_2_O ultrathin nanoslices (~1 nm) and single crystal (NH_4_)(Ni,Co)PO_4_·0.67H_2_O were synthesized through a mild hydrothermal sacrificial template method. The mixed phases present a high specific capacitance of 1128 F g^−1^ at a current density of 0.5 A g^−1^. An asymmetric supercapacitor is fabricated using this hybridized material as positive electrode, and a hierarchical porous carbon previously reported^28^,^29^ as negative electrode. The asymmetric supercapacitor exhibits high energy density of 35.3 Wh kg^−1^ at low power density of 101 W kg^−1^, and still holds 30.9 Wh kg^−1^ at 4400 W kg^−1^, accompanied with excellent cycling stability (95.6% capacitance retention after 5000 charge-discharge cycles).

## Experimental section

### Materials

Reagents nickel nitrate hexahydrate (Ni(NO_3_)_2_•6H_2_O), cobalt nitrate hexahydrate (Co(NO_3_)_2_•6H_2_O), ammonium phosphate ((NH_4_)_2_HPO_4_), potassium hydroxide (KOH), ammonia (NH_3_•H_2_O), polytetrafluoroethylene (PTFE, 10 wt.% water suspension), and absolute ethanol were commercially available with analytical grade. All stock solutions used in this work were prepared with deionized water.

### Precursor-directed synthesis of NH_4_-Ni-Co phosphate

Nickel cobalt hydroxide (Ni,Co-OH) hexagonal nanoplates were first synthesized via a facile hydrothermal procedure (see Supplementary Material for detailed information). 0.186 g (2 mmol) of the as-obtained precursor was dispersed into 50 mL of deionized water through ultrasonication and magnetic stirring to form a uniform suspension. Subsequently, 0.264 g (2 mmol) of ammonium phosphate ((NH_4_)_2_HPO_4_) was dissolved in 20 mL of deionized water, the resulted solution was then added into the above suspension dropwisely. After stirring for 10 mins, the as-obtained material was transferred into a 100 mL Teflon-lined stainless-steel autoclave. The autoclave was heated up to 120 °C and kept there for 24 h and then cooled to room temperature naturally to form a dark pink precipitate. After thoroughly washing with deionized water and ethanol, the final product was dried in vacuum oven at 80 °C for 12 h and denoted as NH_4_CoNiP. For comparison, another two controlled trials by Varing the molar ratio between the NiCo-OH precursor and phosphorus were also prepared (see Supplementary Material for detail).

### Characterizations

Powder X-ray diffraction (XRD) patterns were recorded on an X-ray diffractometer (Rigaku, λ = 1.5418 Å). Field emission scanning electron microscopy (FESEM) images and energy dispersive X-ray spectra (EDX) were taken with a Hitachi SU-8030 field emission scanning electron microscope at the acceleration voltage of 15 kV, and further confirmed on transmission electron microscopy (TEM) (Hitachi-7650, 100 kV 10 μA) equipped with selected area electron diffraction (SAED) system. Atomic force microscopy (AFM) was measured on a Veeco DI Nano-scope MultiMode VIII system.

### Electrochemical measurement

The electrochemical performance of as prepared samples were tested firstly in a three-electrode system. The working electrode was made from mixing the products of NH_4_-Co-Ni phosphate, acetylene black, and polytetrafluoroethylene (PTFE, 1 wt.% water suspension) binder with a weight ratio of 85:10:5, coating onto a nickel foam current collector, vacuum drying 12 h at 80 °C, and then pressing under the pressure of 10 MPa. Each electrode contained about 3.6 mg of electroactive materials with geometric surface area of 1 cm^2^. A platinum plate and Hg/HgO (1 M KOH) electrode was used as the counter and reference electrodes, respectively. The asymmetric supercapacitor was then assembled with a hierarchical porous carbon (HPC) derived from Artemia cyst shell^23^,^24^ (see supplementary material for preparation method) as the active material for negative electrode, and NH_4_CoNiP for the positive electrode respectively. All electrochemical tests were performed in 6M KOH aqueous electrolyte.

Cyclic voltammetry (CV) and the electrochemical impedance spectra (EIS) were tested on a CHI660c electrochemical workstation (Chenhua, China). Current charge-discharge test was performed on a LAND battery program-control test system (Land, CT2001A, China). The electrode capacitance was calculated from the discharge curve according to the following equation:


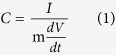


where I (A) is the discharge current, m (g) is the mass of active materials, and (dV)/(dt) (V s^−1^) is the gradient of discharge curves. The power density (P) and the energy density (E) of supercapacitors were calculated according to the following equations


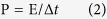



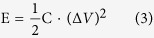


where C is the specific capacitance. ΔV (V) is the potential window, Δt is the discharge time consumed in the potential range of ΔV.

## Results and Discussion

**Figure 1:**
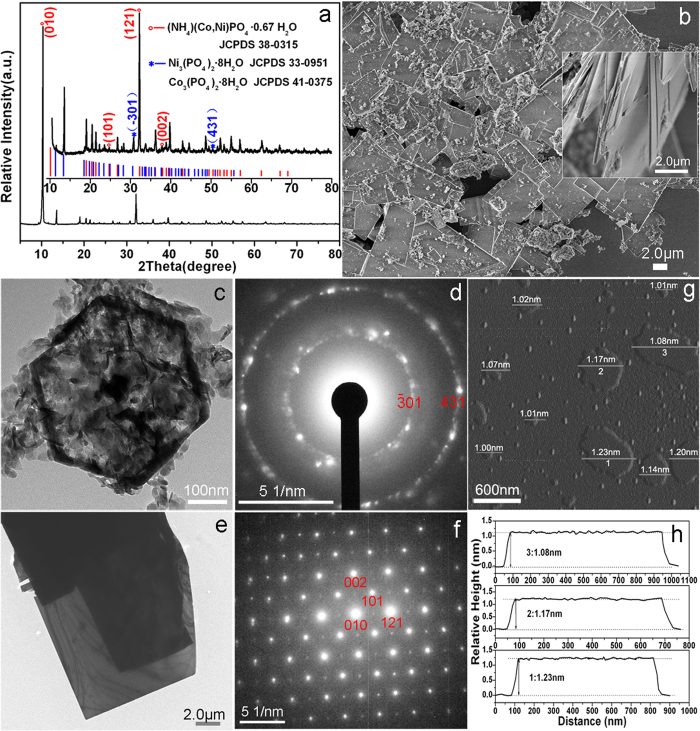
XRD patterns (inset: amplified XRD patterns for 2θ between 10 ~ 80°) (**a**), FESEM image (inset: enlarged view) (**b**), TEM images of hexagonal structure (**c**), large rectangular platelet (**e**) and their SAED patterns (**d,f**), Height profiles derived from the atomic force microscopy (AFM) image of single-layered (NH_4_)(Ni,Co)PO_4_·0.67H_2_O slab in (**g**) and the numbers 1 to 3 in (**g**) correspond to 1 to 3 in (**h**).

The XRD pattern in Fig. 1a can be readily indexed to (NH_4_)(Ni,Co)PO_4_·0.67H_2_O (JCPDS No. 38–0315), and (Co,Ni)_3_(PO_4_)_2_·8H_2_O phase (refers to (Co)_3_(PO_4_)_2_·8H_2_O (JCPDS No. 41–0375), (Ni)_3_(PO_4_)_2_·8H_2_O (JCPDS No. 33-0951), respectively), illustrating the mixed phases of the as prepared sample. In addition, FESEM image (Fig. 1b) also shows two different kinds of morphologies (see inset for the enlarged view), including hexagonal hollow nanoplates and rectangular shaped microplatelets. The hollow hexagonal structure is composed of ultrathin nanoslices (Fig. 1c), which is indexed as the crystal facets of (−301) and (431) of (Ni,Co)_3_(PO_4_)_2_·8H_2_O phase (Fig. 1d). While the rectangular platelet corresponds to the single crystal phase of (NH_4_)(Ni,Co)PO_4_·0.67H_2_O (Fig. 1e,f). The AFM image and corresponding height profiles in Fig. 1g,h depict smooth 2D nanoslices with an average height of about 1.1 nm, which could be considered as a single-layered (Ni,Co)_3_(PO_4_)_2_·8H_2_O, of which the d spacing along the b-axis direction is 0.66 nm.

**Figure 2:**
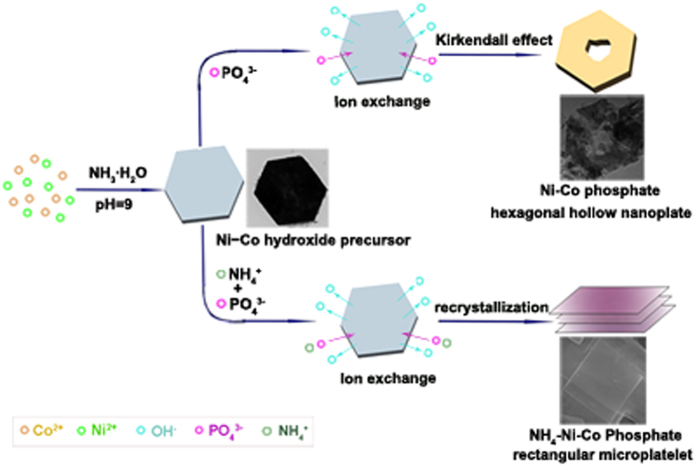
Illustration of the possible structure evolution mechanism.

The formation of hexagonal hollow structure should be attributed to the ion exchange reactions between PO_4_^3−^ and OH^−^ ions based on the Kirkendall effect as reported in other systems^30^. Generally, materials with lower Ksp values are thermodynamically more stable than those with higher Ksp values^31^. In this work, the magnitude of solubility product constant (Ksp) of nickle cobalt hydroxide and phosphate is about 10^−15^ and 10^−31 ^32, respectively. The reaction between PO_4_^3−^ ions and precursor nanoplates results in the production of a layered (Ni,Co)_3_(PO_4_)_2_·8H_2_O nanoslices on the surface of Ni,Co-OH nanoplates. With increasing reaction time, PO_4_^3−^ ions would penetrate into the nickel cobalt hydroxide nanoplates, the direct conversion of the precursor core to the (Ni,Co)_3_(PO_4_)_2_·8H_2_O shell is then obstructed by the nanoslice layers, therefore the further reaction will continue by the diffusion of PO_4_^3−^ ions through the interfaces^33^,^34^. However, due to an imbalance of ion exchange, the outward diffusion rate of the hydroxyl is faster than the inward transport rate of PO_4_^3−^ ions, the (Ni,Co)_3_(PO_4_)_2_·8H_2_O shell will therefore increase, and the precursor core will decrease gradually^35^. Finally, (Ni,Co)_3_(PO_4_)_2_·8H_2_O hollow nanostructures would be obtained. The whole process is illustrated schematically in Fig. 2. Note that, NH_4_^+^ ions can also participate in the reaction by forming (NH_4_)(Ni,Co)PO_4_·0.67H_2_O nanosclies. However, according to the experimental results, there is a large portion of (Ni,Co)_3_(PO_4_)_2_·8H_2_O produced. This should be due to that, the reaction took place in a slightly alkaline environment, of which the pH value was measured as ∼8. In this condition, a big part of NH_4_^+^ ions will go through a hydrolysis process to form aqueous ammonia, while only small amount of NH_4_^+^ ions remain to form (NH_4_)(Ni,Co)PO_4_·0.67H_2_O precipitates. To further verify this mechanism, the phases and morphologies of the control samples with different composition were also investigated and shown in Figs. S2 and S3. Pure (NH_4_)(Ni,Co)PO_4_·0.67H_2_O can only be obtained with excessive amount of ammonium phosphate added (Supplementary Fig. S3b,d).

**Figure 3:**
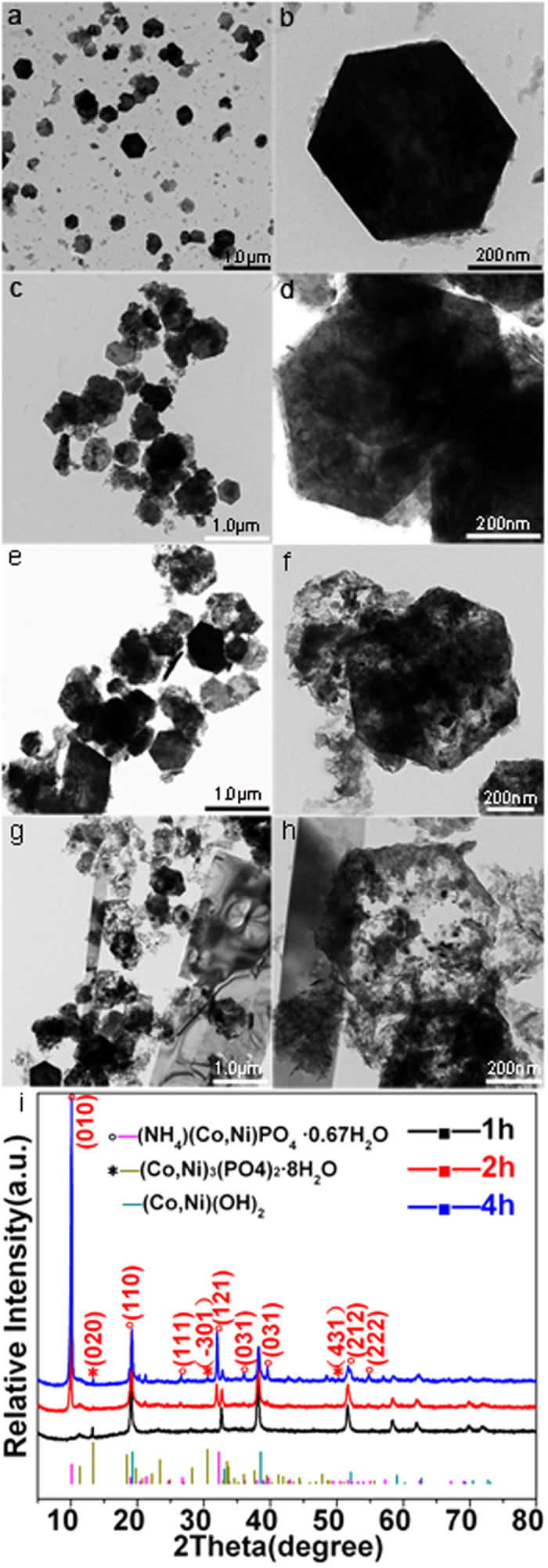
TEM images of samples (**a,b**) Ni,Co-OH precursor, and products at different reaction stage (**c,d**) 1 h, (**e,f**) 2 h, (**g,h**) 4 h; (**i**) XRD patterns of products at different reaction stage.

To monitor the formation process of this novel composite structure, time-dependent morphology and phase evolution were examined by TEM and XRD as shown in Fig. 3. It can be seen from Fig. 3a,b, the NiCo-hydroxide precursor is composed of dense hexagonal structure with smooth surface. After reaction with ammonium phosphate ((NH_4_)_2_HPO_4_) at 120 °C for 1 h, the products still present hexagonal structure but with much rougher surface, and small nanoslices were observed around the hexagonal nanoplates, indicating the occurrence of ion exchange (Fig. 3c,d). The corresponding XRD patterns demonstrate that these hexagonal nanoplates are mainly NiCo-hydroxide precursors, and only a few extremely weak peaks (e.g. the (020) facet at 2θ of 13.3°) of (Ni,Co)_3_(PO_4_)_2_·8H_2_O is observed (Fig. 3i). When prolonging the reaction duration to 2 h, the products still maintain the original hexagonal structure, except that the nanoplates start to become thinner with increased surface roughness, indicating further ion exchange reaction on the precursor surface (Fig. 3e,f). Two obvious diffraction peaks at 10.1°, 32° and one weak peak at 13.3° appear in the XRD patterns (Fig. 3i), which can be indexed to the (010), (121) facets of (NH_4_)(Ni,Co)PO_4_·0.67H_2_O and (020) facets of (Ni,Co)_3_(PO_4_)_2_·8H_2_O, respectively. With further increasing the reaction time to 4 h, hollow hexagonal structure is observed, accompanied with the simultaneous formation of microsized platelets, indicating a process of dissolution recrystallization of (NH_4_)(Ni,Co)PO_4_·0.67H_2_O single crystal. The corresponding XRD pattern coincides with the mixed phase of hexagonal hollow structured (Ni,Co)_3_(PO_4_)_2_·8H_2_O with the crystal planes of (020), (−301) and (431) as well as phase of oblong microplatelet structured (NH_4_)(Ni,Co)PO_4_·0.67H_2_O with the crystal planes of (010) and (121) (Fig. 3i). Therefore, the formation of such novel hybridized structure can be explained by the repeated anion exchange and the followed dissolution recrystallization process taking place on the pre-formed precursor surface.

**Figure 4:**
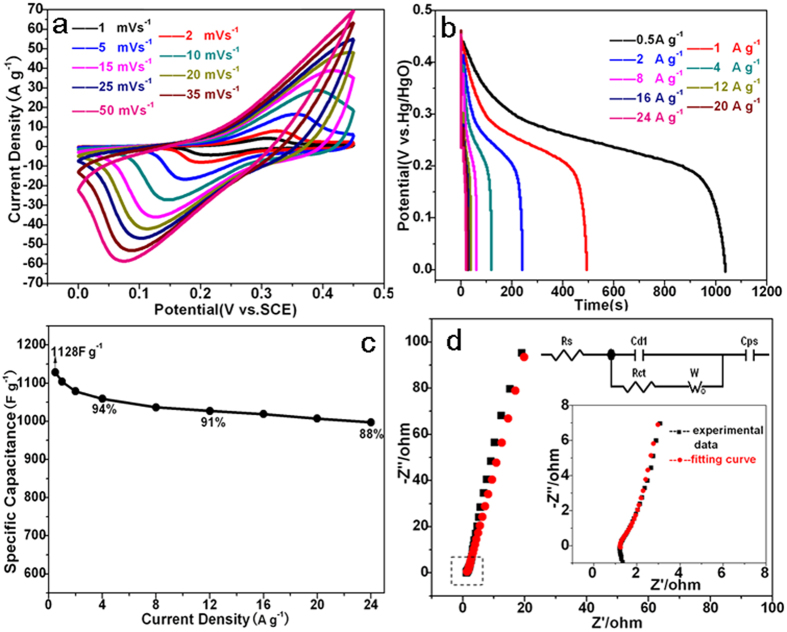
(**a**) CV curves at different scan rates, (**b**) GCD curves at different current densities, (**c**) specific capacitance at different current densities, (**d**) the Nyquist plots and the fitting curve.

**Figure 5:**
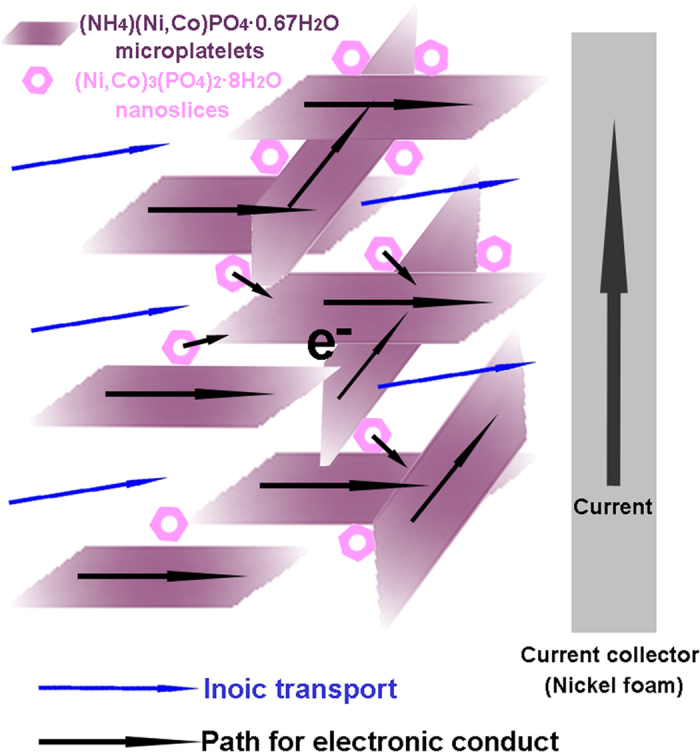
Illustration of the ionic and electronic conduction mechanism for the hybrid structure.

Electrochemical performance of the hybridized material was tested with a three-electrode system in 6 M KOH within a potential window of 0 to 0.45 V. Figure 4a shows the typical CV curves from 1 to 50 mV s^−1^. Strong peaks around 0.3 to 0.4 V and 0.15 to 0.25 V are observed from all the samples, suggesting the reversible Faradaic redox processes of Co^2+^/Co^3+^ and Ni^2+^/Ni^3+^ in KOH solution^35^. Figure 4b shows the discharge curves of the as prepared samples at different current densities, which is in good agreement with CV results. The specific capacitances (SC) at different current densities are calculated from the discharge curves and plotted in Fig. 4c. The hybridized material delivers a high SC of 1128 F g^−1^ at 0.5 A g^−1^, which remains 997 F g^−1^ even at high current density of 24 A g^−1^, suggesting a high capacitance retention of 88.4%. To identify the electrical conductivity of electrode, the EIS spectrum was measured within the frequency range of 10 kHz to 0.1  Hz and shown in Fig. 4d. The Nyquist plot consists of a semicircle accompanied by a straight line at the low frequency region, which correspond to the electrochemical process and mass transfer process, respectively^36^,^37^. An equivalent circuit constituted of an equivalent series resistance (Rs), a charge transfer resistance (Rct), a double layer (C_dl_) and a pseudocapacitive element (Cps), and Warburg impedance (W) was constructed, and the fitting curve matches well with the experimental data. The Rs and Rct values are calculated as 1.29 and 1.38 Ω, respectively, indicating low contact resistance and low charge transfer resistance of the electrode. This could be attributed to that, this novel hybrid material provides a suitable structure for both ionic transport and electronic conduction. The (NH_4_)(Ni,Co)PO_4_·0.67H_2_O microplates offer a continuous electronic conduction path by bridging the (Ni,Co)_3_(PO_4_)_2_·8H_2_O nanoslices, and the channels formed between the microplate and nanoslices facilitate the ionic transportation (Fig. 5). For comparison, the electrochemical performance of the control samples was also studied (Fig. S4). Sample NH_4_CoNiP_0.5_ presents good SC (Fig. S4c) at low current densities, but poor rate performance, which should be attributed to its poor electric conductivity (Fig. S4d). The comparison of the calculated resistance values (Table S1) further confirmed this assumption. On the contrary, sample NH_4_CoNiP_3_ exhibits good rate performance but much lower SC.

Therefore, the synergistic effect of both structures endows NH_4_NiCoP with superior capacitance behavior. To further understand the energy storage behavior of this unique hybridized structure, a possible mechanism is proposed. The redox reaction of (NH_4_)(Co,Ni)PO_4_·0.67H_2_O and (Ni,Co)_3_(PO_4_)_2_·8H_2_O follows eq. 4 and eq. 5, respectively:


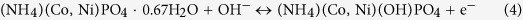



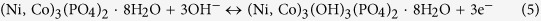


**Figure 6:**
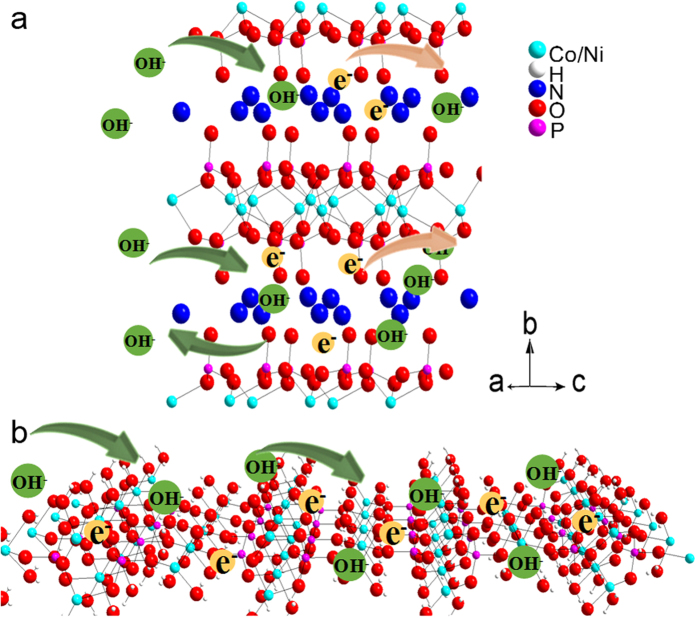
Schematic Illustration of (**a**) the intercalation/deintercalation of hydroxyl ions, (**b**) surface redox reaction.

The single layer nanoslices from the hexagonal hollow (Ni,Co)_3_(PO_4_)_2_·8H_2_O can provide much more electroactive sites for surface redox reaction^26^ than the bulk material, as illustrated in Fig. 6a. While the lamellar structured single crystal (NH_4_)(Ni,Co)PO_4_·0.67H_2_O with large layer distance of 0.875 nm is favorable for the diffusion of ions and electrons, which endows the samples with enhanced electric conductivity and fast ions in and out, and hence enable the intercalation and de-intercalation process of hydroxyl ions between the atomic layers (Fig. 6b). Therefore, the hybrid materials consisted of both structures would benefit from the synergistic effect of both reaction mechanism, and exhibits enhanced capacitance behavior.

**Figure 7:**
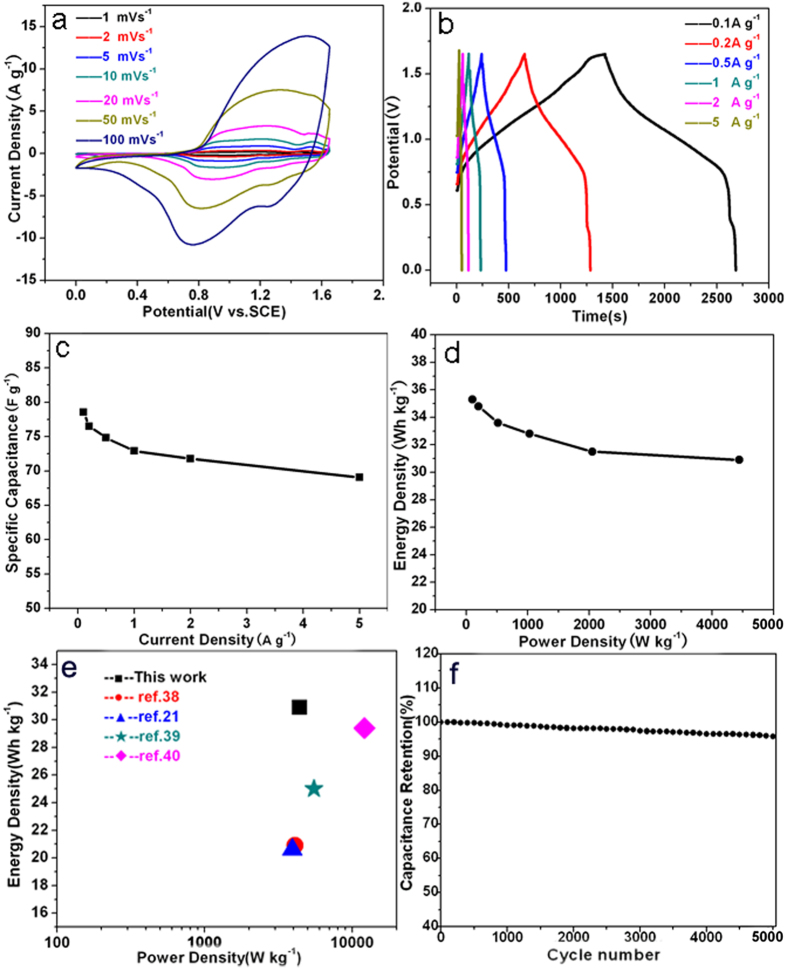
Electrochemical performance of the NH_4_CoNiP//HPC asymmetric supercapacitor: (**a**) CV curves with a voltage of 1.65 V at different scan rates, (**b**) GCD plots at different current densities, (**c**) Specific capacitances as a function of discharge current densities, (**d**) Ragone plot, (**e**) a comparison among as-made Ni,Co phosphate//HPC and previously reported ASCs, (**f**) cycling performance at a constant current density of 1 A g^−1^.

In order to certify the practical application of the hybridized material for energy storage, an asymmetric supercapacitor cell was assembled with HPC (see supplementary material (Fig. S5) for electrochemical performance of HPC) as the negative electrode and NH_4_CoNiP as the positive electrode, which is denoted as NH_4_CoNiP//HPC. The different operation voltages of the NH_4_CoNiP electrode (0 ~ 0.45 V) and HPC electrode (−1.2 ~ 0 V) indicate a perfect match on the potential windows for an asymmetric supercapacitor (Fig. S5f). The loading amount of positive and negative active materials were decided following the relationship of


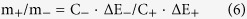


where C_+_ and C_−_ represent the specific capacitance values of the positive and negative electrodes, respectively, and ΔE is the potential range (See supplementary materials for detailed information). Fig. 7a shows the CV curves of the asymmetric supercapacitor cell with a voltage range of 1.65 V at different scan rates. It is noticed that the CV curve in high scan rate of 100 mV s^−1^ retains the shape at low scan rates, indicating the high rate-capacity of NH_4_CoNiP//HPC supercapacitors. Fig. 7b shows the GCD curves of the asymmetric supercapacitor cell at various current densities in a voltage window from 0 to 1.65 V with a nearly symmetrical shape, indicating good electrochemical supercapacitor characteristics. The SC was calculated from the discharge curves based on the mass of active materials from both electrodes as shown in Fig. 7c. Fig. 7d displays a high energy density of 35.3 Wh kg^−1^ at a power density of 101 W kg^−1^. And the energy density remains 30.9 Wh kg^−1^ at a high power density of 4400 W kg^−1^, which is superior to those asymmetric supercapacitors reported in literature^21^,^38^,^39^,^40^ (Fig. 7e), implying the promising potential application in energy storage devices. The long cycle performance of the asymmetric supercapacitor was tested at a current density of 1 A g^−1^ (Fig. 7f), 95.6% capacitance retention remains after 5000 charge-discharge cycles, which is significantly enhanced as compared to those phosphates reported previously^22^,^23^.

## Conclusions

In this work, the NH_4_-Co-Ni triple cation phosphates with controlled structures have been firstly synthesized through a mild hydrothermal method. The formation mechanism of the unique hybridized structure is discussed in detail. Attributed to the synergistic effect, this novel hybrid exhibits a high specific capacitance of 1128 F g^−1^ at a current density of 0.5 A g^−1^, and remains 997 F g^−1^ at 24 A g^−1^. The asymmetric supercapacitor cell based on this material exhibits a high energy density of 30.9 Wh kg^−1^ at 4400 W kg^−1^ along with excellent cycling stability (95.6% capacitance retention after 5000 cycles). Such results suggest that our material is promising to be a novel kind of electrode materials with high electrochemical properties for the practical application in supercapacitors.

## Additional Information

**How to cite this article**: Zhao, Y. *et al*. Hybridized Phosphate with Ultrathin Nanoslices and Single Crystal Microplatelets for High Performance Supercapacitors. *Sci. Rep*. **6**, 17613; doi: 10.1038/srep17613 (2016).

## Supplementary Material

Supplementary Information
