# Supplementary material for: Hybridized Phosphate with Ultrathin Nanoslices and Single Crystal Microplatelets for High Performance Supercapacitors
Source: Sci Rep. 2016 Feb 1;6:17613. doi: 10.1038/srep17613 (PMC4735281; doi:10.1038/srep17613)
Supplement: Supplementary Information [file srep17613-s1.doc]

**Hybridized Phosphate with Ultrathin Nanoslices and Single Crystal Microplatelets for High Performance Supercapacitors**

*Yufeng Zhao^1*^, Zhaoyang Chen^1^, Ding-Bang Xiong^2^, Yuqing Qiao^1^, Yongfu Tang^1^, Faming Gao^1**^*

*^1^Key Laboratory of Applied Chemistry, Yanshan University, Qinhuangdao 066004, China. Corresponding Email:^1*^ yufengzhao@ysu.edu.cn; (Y. Zhao)*

*^1**^ fmgao@ysu.edu.cn (F. Gao)*

*^2^State Key Laboratory of Metal Matrix Composites, Shanghai Jiao Tong University, Shanghai 200240, China*

**Experiment**

**Preparation of Ni,Co hydroxide precursor**

To prepare Ni,Co-OH precursor, 0.50 g Ni(NO_3_)_2_·6H_2_O (1.7 mmol) and 0.50 g Co(NO_3_)_2_·6H_2_O (1.7 mmol) were dissolved in 70 mL of deionized water, magnetic stirred for a few minutes to obtain a clear solution, and then 7.5 ml ammonia solution (2 mol/L) was dropwisely added. After being stirred for 30 mins, the solution was transferred into a 100 mL Teflon-lined stainless-steel autoclave and heated at 120 °C for 4 h. After cooling to room temperature naturally, the grey precipitate was collected and successively washed with deionized water/ethanol for several times and then dried in vacuum oven at 80 °C for 12 h.

**Preparation of control samples**

The other two control samples were prepared with molar ratio *m*(Ni,Co-OH): *m*((NH_4_)_2_HPO_4_) equals to 0.5 and 3 respectively. Specifically, 0.132 g (1 mmol) and 0.792 g (6 mmol) of ammonium phosphate were added with other conditions unchanged, which are denoted as NH_4_CoNiP_0.5_ and NH_4_CoNiP_3_, respectively.

**Preparation of hierarchical porous carbon (HPC)**

HPC was prepared as reported previously [1]. Artemia cyst shells were cleaned with deionized water, dried, and then ball-milled for 6 h at a speed of 300 rpm. The ball-milled Artemia cyst shells were heated to 300^o^C at Ar gas atmosphere with a heating rate of 5^o^C min^-1^ and maintain for 3 h for pre-carbonization, then heated up to 700^o^C at 5^o^C min^-1^ and kept for 4 h. The obtained products were sonicated in 67 wt% HNO_3_, then washed with deionized water and dried at 80^o^C for 12 h, the final product is named as HPC.

**Supporting Figures**

**
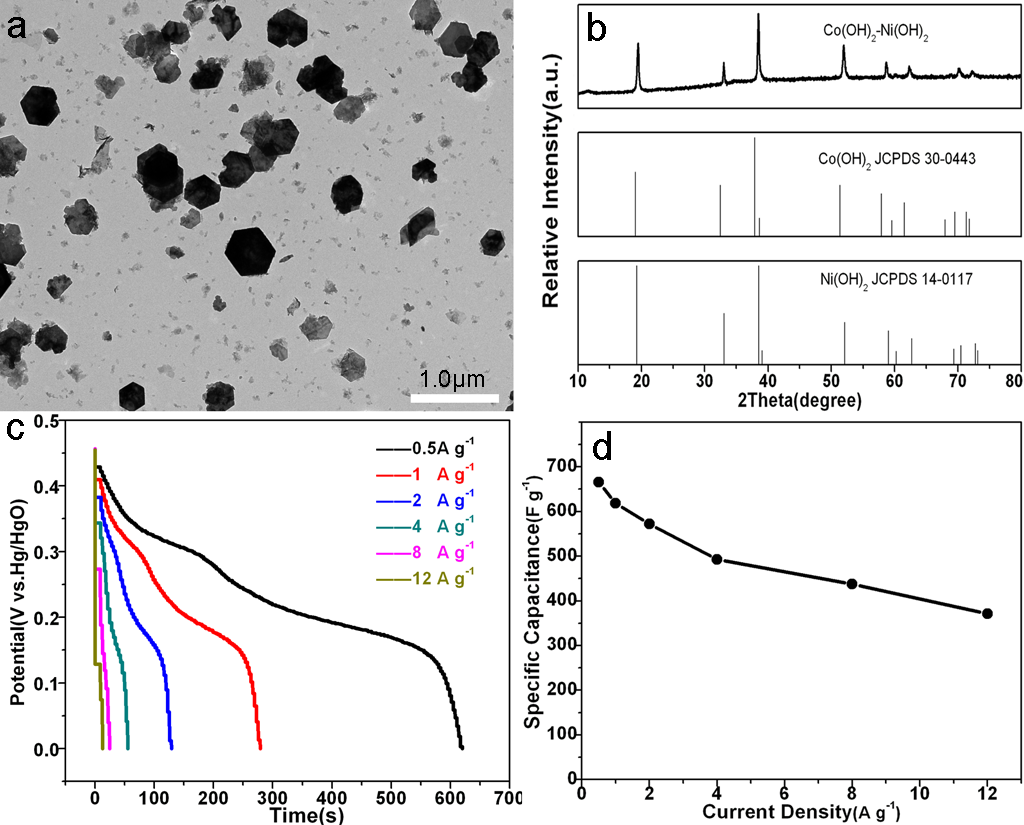
Figure S1**. TEM images (a), XRD patterns (b), galvanostatic discharge curves (c), specific capacitances at different current densities (d) for the Ni,Co-OH precursor.

The morphology of the as prepared Ni,Co-OH precursor is shown in **Figure S1a,** the XRD pattern in **Figure S1b** can be readily indexed to Co(OH)_2_ (JCPDS No. 30-0443), and Ni(OH)_2_ (JCPDS No. 14-0117) phase. Electrochemical performance of the as prepared precursor was tested with a three-electrode system in 6M KOH within a potential window of 0 to 0.45 V. **Figure S1c** shows the discharge curves of the as prepared samples at different current densities, the specific capacitances (SC) at different current densities are calculated from the discharge curves and plotted in **Figure S1d**. The precursor delivers a SC of 666 F g^-1^ at 0.5 A g^-1^, which remains 371 g^-1^ at 12 A g^-1^, suggesting a capacitance retention of 55.7%.

**
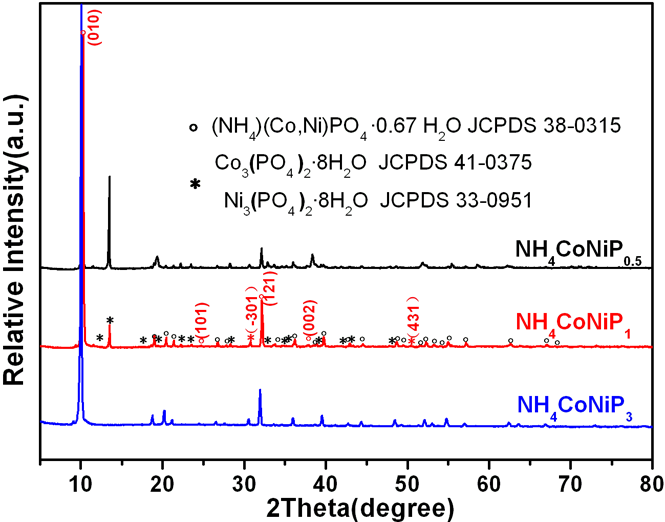
**

**Figure S2**. XRD patterns of the as prepared samples.

**Figure S2** shows the XRD patterns of the as prepared samples. The characteristic peaks of (Co,Ni)_3_(PO_4_)_2_·8H_2_O, take the peak at 2θ of 13.3° (020) as an example, which is strong and sharp for sample NH_4_CoNiP_0.5_, significantly reduced in the pattern of sample NH_4_CoNiP, and finally disappeared in the pattern of NH_4_CoNiP_3_. At the same time, the characteristic peaks of (NH_4_)(Ni,Co)PO_4_·0.67H_2_O, for example 2θ of 10.1° (010) and 32.2° (121) are comparatively enhanced. This implies that with the increasing amount of ammonium phosphate, more (NH_4_)(Ni,Co)PO_4_·0.67H_2_O were formed. With overamount of ammonium phosphate added for sample NH_4_CoNiP_3_, pure (NH_4_)(Ni,Co)PO_4_·0.67H_2_O phase were finally obtained.


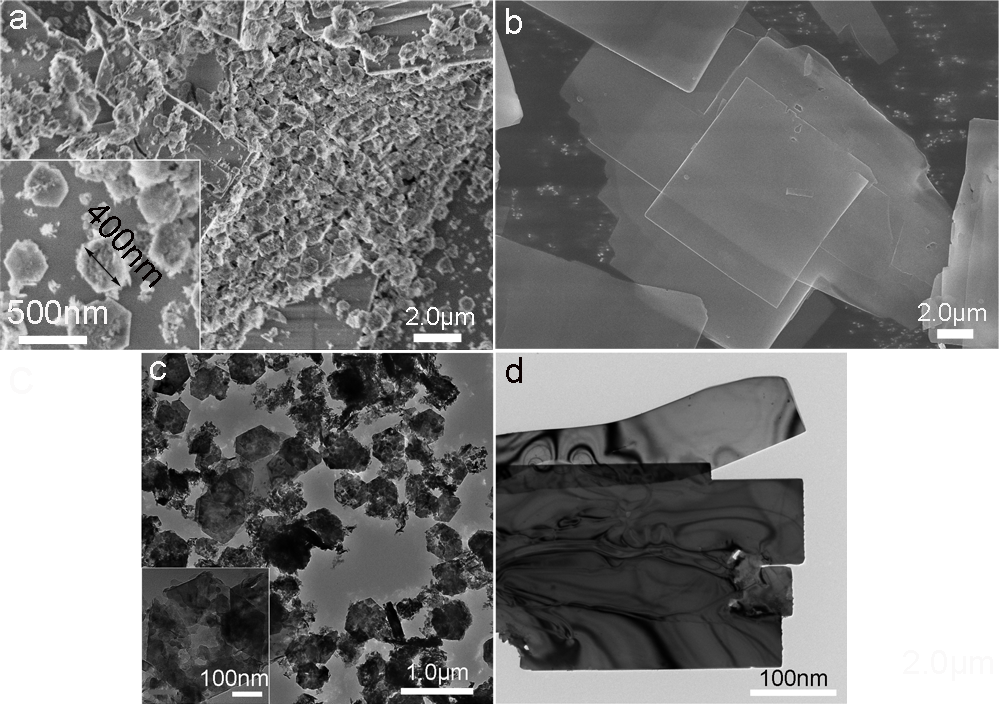


**Figure S3.** FESEM images of NH_4_CoNiP_0.5_ (a), NH_4_CoNiP_3_ (b), and TEM images of NH_4_CoNiP_0.5_ (c), NH_4_CoNiP_3_ (d).


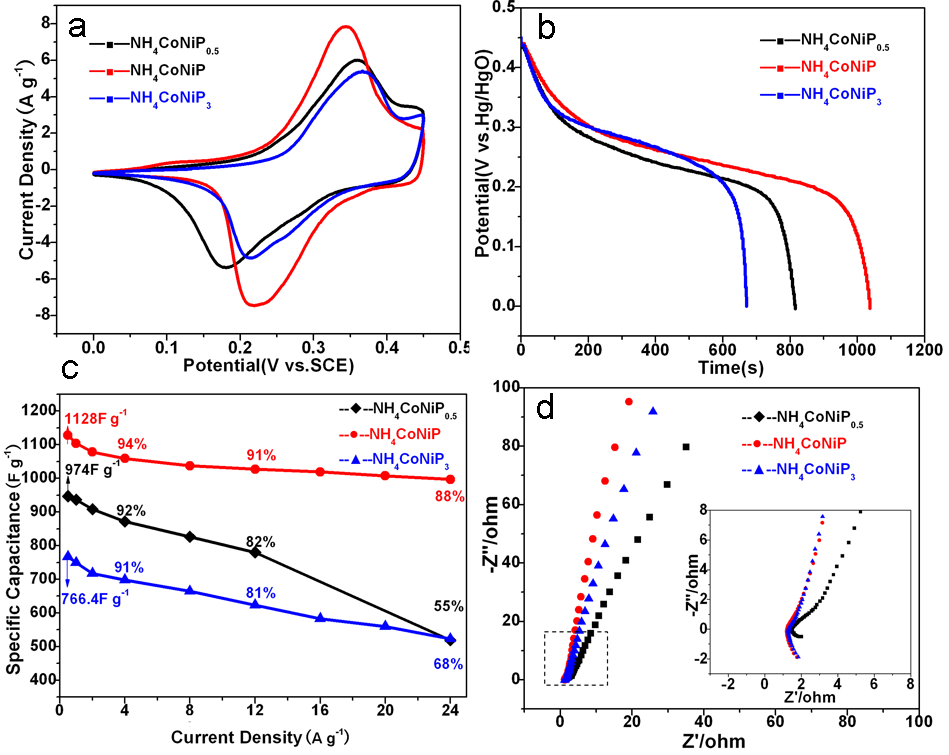


**Figure S4**. CV curves of at 2 mV s^-1^ (a), galvanostatic discharge curves (b) at 0.5 A g^-1^ specific capacitances (c) at different current densities, and Nyquist plots (d).


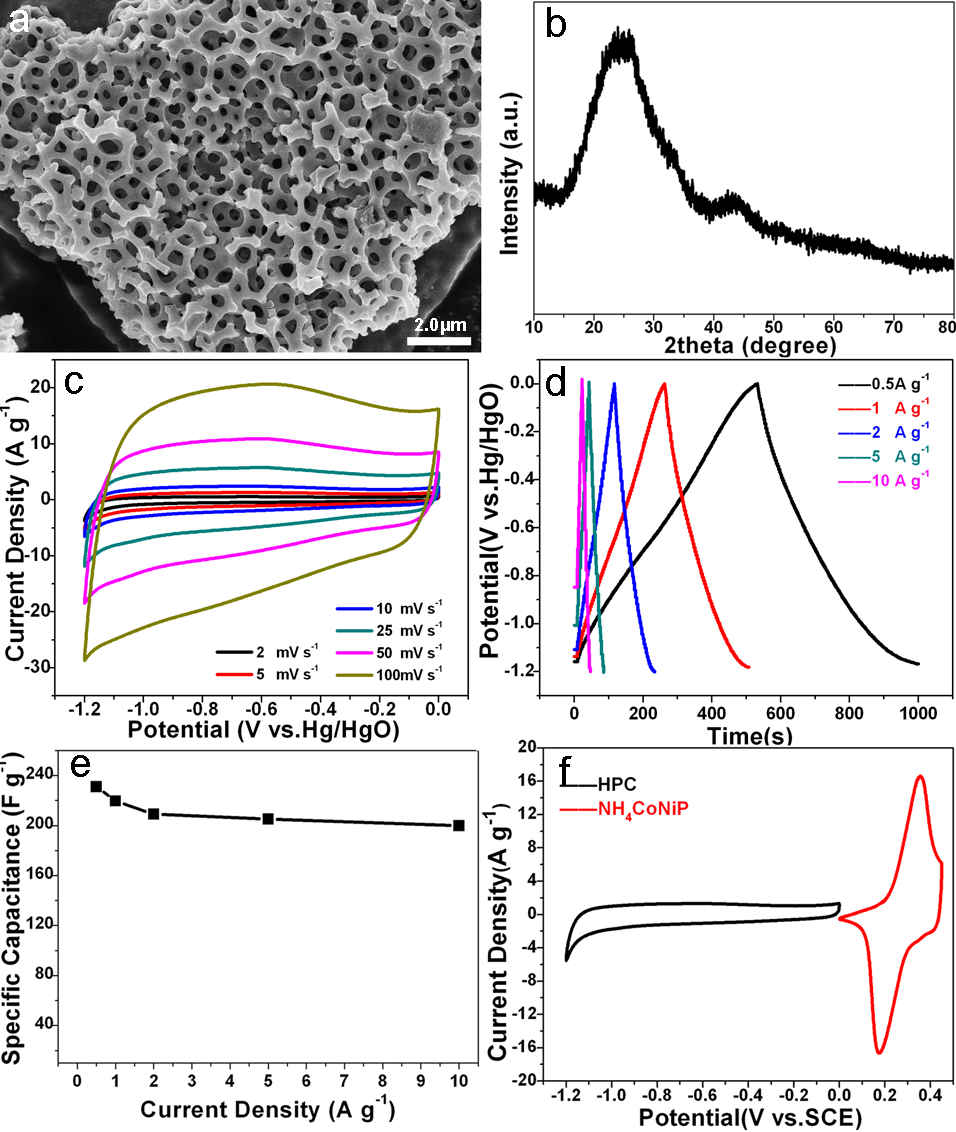


**Figure S5**. FESEM image (a), XRD pattern (b), CV curves at different scan rates (c), GCD curves at different current densities (d), SC as a function of discharge current densities (e) of HPC, and CV curves of NH_4_CoNiP and HPC at 5 mV s^-1^ (f).

**Figure S5a** shows the FESEM image of the HPC derived from Artemia cyst shells, the hierarchical porous structure of the AS is well preserved. **Figure S5b** shows the XRD patterns of the as prepared HPC. Broad bumps at a 2θ of about 26.1° and 43° corresponding to the (002) and (100) reflections of carbon are observed, indicating the mainly amorphous characteristics of the HPC. **Figure S5c** shows CV curves of HPC electrodes tested with a three electrode system in the potential range of -1.2~0 V with scan rates from 2 to 100 mV s^-1^ in 6M KOH. **Figure S5d** shows GCD curves of HPC at different current densities from 0.5~10 A g^-1^. The corresponding SC as a function of discharge current densities is calculated**,** the HPC delivered a SC of 230 F g^-1^ at 0.5 A g^-1^ which remains 200 F g^-1^ at 10 A g^-1^ as shown in **Figure S5e**. **Figure S5f** shows the CV curves of HPC and the as prepared NH_4_CoNiP at the scan rate of 5 mV s^-1^, the different operation voltages of the NH_4_CoNiP electrode (0~0.45 V) and HPC electrode (-1.2~0 V) indicate a perfect match on the potential windows for an asymmetric supercapacitor. Following the relationship of Δ*Q_+_* =Δ*Q*- , the mass ratio of positive and negative electrodes ( m_+_/m-), can be calculated by equation m_+_/m- = C-•ΔE-/C_+_•ΔE_+_, where C_+_ and C- represent the specific capacitance values of the positive and negative electrodes, respectively, and ΔE is the potential range. The theoretically optimal mass ratio between NH_4_CoNiP and HPC electrodes should be m_+_/m- ≈ 0.53 in the two electrode cell. Based on this we fabricated an asymmetric supercapacitor cell using NH_4_CoNiP as the positive electrode, HPC as the negative electrode, and 6M KOH as the electrolyte. The loading amount of positive and negative active materials is 2.0 mg cm^-2^ and 3.8 mg cm^-2^, respectively, with the total loading of 5.8 mg cm^-2^.


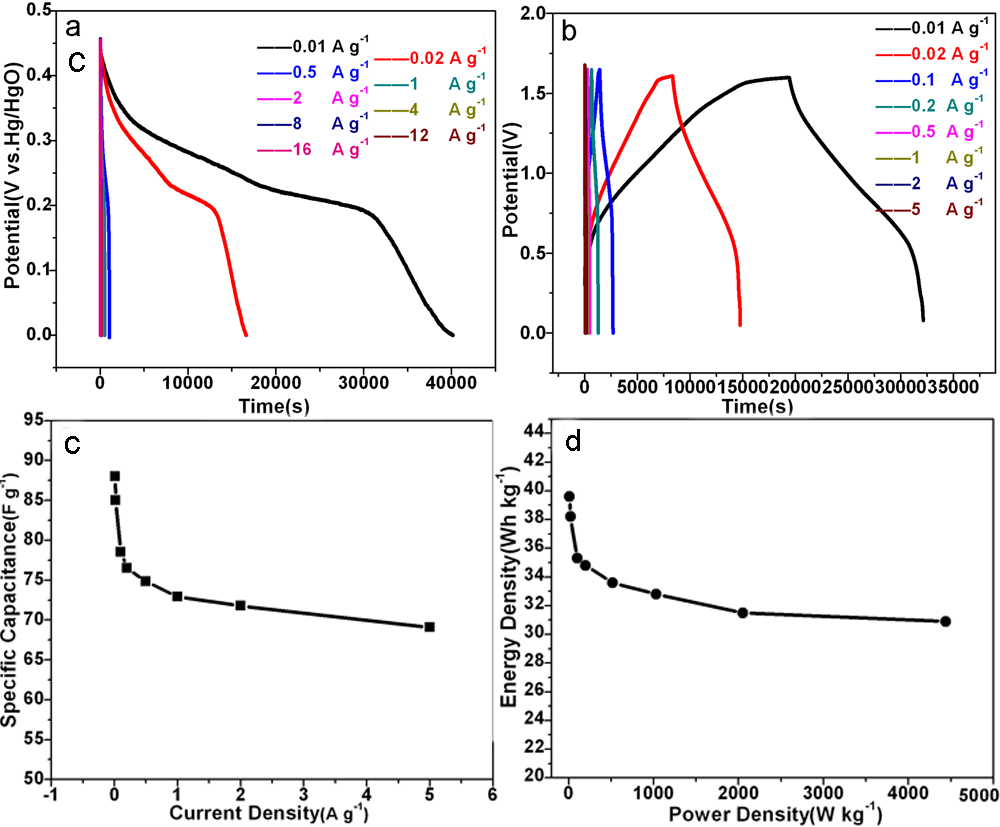


**Figure S6.** (a) GCD curves from 0.01 to 16 A g^-1^ for a three-electrode system, (b) GCD curves from 0.01 to 5 A g^-1^ for ASC, (c) specific capacitance from 0.01 to 5 A g^-1^ for ASC, (d) Ragone plot from 11 to 4400 W kg^-1^

The specific capacitances (SC) at very low current densities of 0.01 and 0.02 A g^-1^ were tested and calculated (**Figure S6a**), which are 1293 F g^-1^ and 1157 F g^-1^ at 0.01 A g^-1^ and 0.02 A g^-1^ respectively. Therefore, a high capacitance retention of 77% from 0.01 to 24 A g^-1^ is achieved, indicating good rate performance for the three-electrode system. GCD curves for the asymmetric supercapacitor cell (ASC) NH_4_CoNiP//HPC at very low current densities were also tested (**Figure S6b)**, the SC values were calculated as 88 F g^-1^ at 0.01 A g^-1^ and 85 F g^-1^ at 0.02 A g^-1^ (**Figure S6C**). The energy density at very small power density (11 W kg^-1^) is 39.6 Wh kg^-1^, indicating a good rate performance with a retention of 78% from 11 to 4400 Wh kg^-1^ (**Figure S6D**).


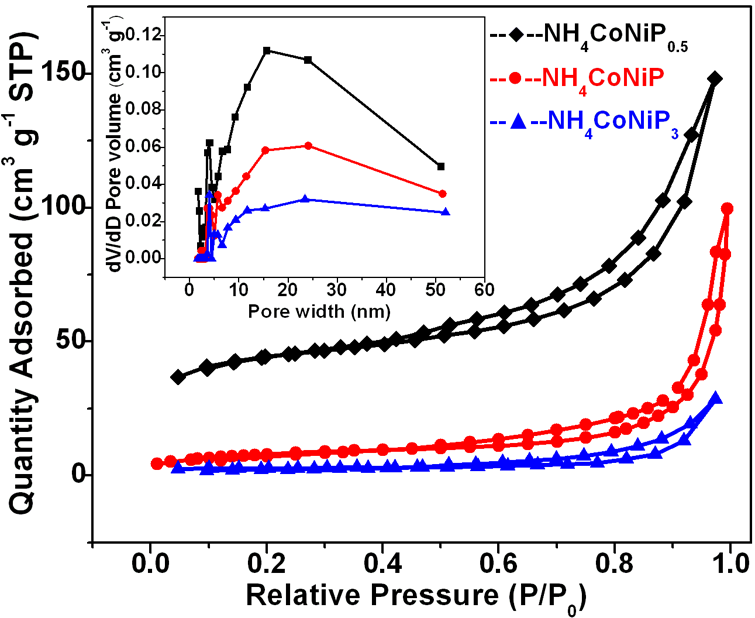


**Figure S7.** N_2_ adsorption-desorption isotherms at 77 K of the three samples.

The nitrogen adsorption/desorportion was carried out using ASAP 2020 HD88 (Micromeritics Instrument Corporation) apparatus at 77 K. The N_2_ adsorption-desorption isotherms were shown in Figure S9. The specific surface area (SSA) and pore size distributions of the materials were calculated using the Brunauer-Emmett-Teller (BET) and Barrett-Joyner-Halenda (BJH) methods, respectively. The BET SSA for sample NH_4_CoNiP_0.5_, NH_4_CoNiP and NH_4_CoNiP_3_ are 43.6, 29.4 and 8.4m^2^ g^-1^, respectively. Considering the maximum specific capacitance 974, 1128 and 766 F g^-1^ for the above samples, there is no obvious correlation between the capacitance behavior and SSA.


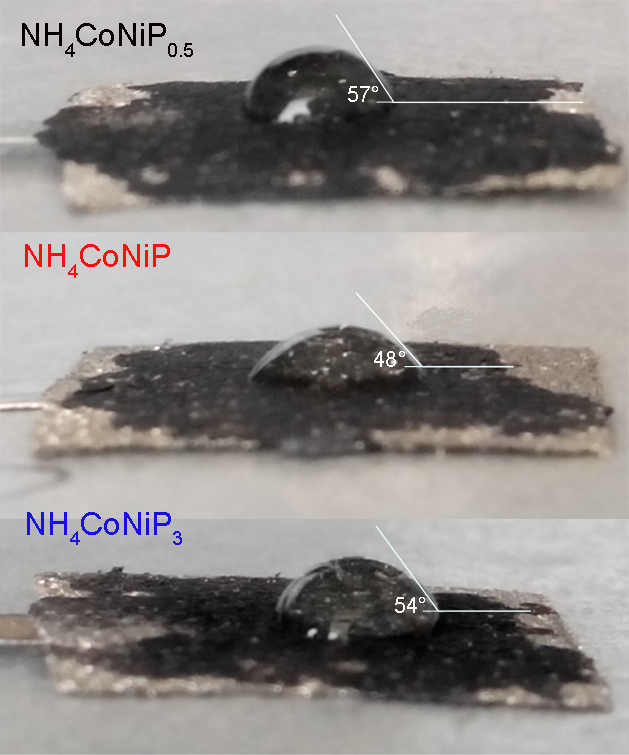


**Figure S8** Contact angle between the as prepared samples with KOH electrolyte

The wettability of the as prepared samples in the KOH electrolyte was also tested as shown in **Figure S8**. The low contact angles indicate good wettability of all the three samples.

**Table S1. Calculated Rs and Rct for the as prepared samples.**

| samples | **Rs (Ω)** | **Rct （Ω）** |
| --- | --- | --- |
| NH_4_CoNiP_0.5_ | 1.89 | 2.74 |
| NH_4_CoNiP | 1.29 | 1.38 |
| NH_4_CoNiP_3_ | 1.37 | 1.94 |

The fitting results for the EIS curves in Figure S4d based on the equivalent circuit model (inset of **Figure 4d**) is listed in **Table S1**, wherein Rs is the bulk solution resistance, Rct is the charge-transfer resistance. Sample NH_4_CoNiP presents both lowest Rs and Rct value as compared to NH_4_CoNiP_0.5_ and NH_4_CoNiP_3_, indicating both low bulk solution resistance and charge transfer resistance for sample NH_4_CoNiP, which facilitates the fast transfer of electrons in the charge-discharge process, giving rise to the high rate capability [2].

**References**

[1] Y.F. Zhao, W. Ran, J. He, Y.F. Song, C.M. Zhang, D.B. Xiong, F.M. Gao, J.S. Wu, and Y.Y. Xia, ACS Appl. Mater. Interfaces, 2015, 7, 1132-1139. 52-59.

[2] H.C. Chen, J.J. Jiang,Y.D. Zhao, L. Zhang, D.Q. Guoand D.D.Xia, J. Mater. Chem. A, 2015, 3, 428-427.
